# Effect of diet video-drama and telephone messages on improving parental knowledge and diet diversity of malnourished children in Kenya: A randomised controlled trial

**DOI:** 10.1371/journal.pgph.0004818

**Published:** 2025-07-09

**Authors:** Beatrice C. Mutai, Fredrick Were, Jalemba Aluvaala, Grace John-Stewart, E. Maleche-Obimbo

**Affiliations:** 1 Department of Paediatrics & Child Health, Faculty of Health Sciences, University of Nairobi, Nairobi, Kenya; 2 Department of Global Health, Medicine, Epidemiology and Paediatrics, University of Washington, Washington State, United States of America; PLOS: Public Library of Science, UNITED STATES OF AMERICA

## Abstract

Severe acute malnutrition (SAM) accounts for 1 million deaths globally each year. Ready-to-use Therapeutic Food (RUTF), recommended for treatment, is often replaced with low-nutrient home foods. We sought to determine the effect of enhanced caregiver counselling, using a dramatized video with contextualized demonstrations of local high-nutrient food (video-drama) and telephone messages on high-nutrient foods (SMS), on children’s dietary diversity scores (DDS), weight gain, and caregiver knowledge. This randomised trial enrolled 213 severely malnourished children and caregivers at Mbagathi Hospital in Nairobi. Children were randomised to 3 study arms: standard of care (SOC) (children received RUTF, caregivers received routine nutrition counselling); intervention arm A (caregivers watched the video-drama at enrolment, 1- and 6-weeks post-enrolment plus SOC); and intervention arm B (caregivers received weekly SMS, watched the video-drama plus SOC). Primary outcome was DDS, secondary outcomes were weight gain and caregiver knowledge. Median DDS, mean rate of weight gain and caregiver knowledge were compared between trial arms using Kruskal-Wallis and ANOVA tests, respectively. Children’s median age at enrolment was 12 months (IQR 8.0, 16.0), 50.7% were female, 74.6% were breastfeeding and 78.9% were on RUTF. Median caregiver age was 28 years (IQR 24.5, 32.0) and 98% were female. Post-intervention, children in arms A and B had significantly higher median DDS at 5 (IQR 4, 5) versus 4 (IQR 3, 5) in SOC arm (p < 0.001)]. Mean caregiver knowledge was significantly higher in arm A (4.53[±1.17)] and arm B (4.27[±1.04]) compared to 3.77(±0.91) in SOC (p < 0.001). Mean rate of weight gain was similar across study arms [7.60 g/kg/day in intervention arms, 7.30 g/kg/day for SOC (p-value 0.31)]. Video-drama enhanced SOC counselling of local high-nutrient foods, improved children’s DDS and caregiver knowledge, but did not improve short-term weight gain. Weekly SMS did not provide additional benefits to the video-drama.

## Introduction

Severe acute malnutrition (SAM) is a leading cause of child mortality in developing countries and accounts for approximately 1 million deaths among children below the age of five years each year globally [1]. According to the Kenya Demographic and Health Survey report of 2022, five percent of children below the age of five years in Kenya suffer from moderate-to-severe acute malnutrition [2]. Studies from Bangladesh and Malawi report an 8–10% mortality rate among children with SAM in the first three months of post-hospital discharge [3,4]. This high mortality rate underscores the need for optimal nutrition and rapid weight gain during recovery. World Health Organization (WHO) recommends Ready-to-Use-Therapeutic-Food (RUTF), a commercially prepared high-protein, high-calorie semi-solid food, for nutritional rehabilitation of children with SAM who are between the ages of 6–59 months [5]. While previous studies in Senegal and Sierra Leone demonstrated good weight gain when children received RUTF within hospital settings, community-based studies conducted in Malawi and Kenya demonstrated poor weight gain among children receiving RUTF at home [6–10]. Children often do not adhere to RUTF prescriptions due to many challenges that include sharing of RUTF within households and sale by caregivers, and continue to receive low-nutrient home foods [11,12]. There is a positive correlation between dietary diversity and micronutrient adequacy of children’s diets as demonstrated in studies conducted in South Africa and Mali [13,14]. WHO categorizes foods into seven groups: cereals, roots, and tubers; legumes and nuts; milk and other dairy products, eggs; meats; vitamin A-rich fruits and vegetables; and other fruits and vegetables (S1 Fig). Dietary diversity score (DDS) is derived through a simple count of the number of food groups consumed, and a higher DDS is associated with higher diet nutrient content. DDS is the WHO-recommended indicator of diet quality in Infant and Young Child Feeding (IYCF) as it is a simple and low-cost method of assessment at the population level [15]. Consumption of foods from four of the seven food groups within 24 hours is the required minimum to ensure a nutritionally adequate diet. Children with SAM continue to receive low-nutrient foods at home, despite the facility-based caregiver counselling on appropriate high-nutrient foods. Mobile health (mHealth) interventions have been shown to effectively improve caregiver knowledge and child-feeding practices in low and middle-income countries. In Kenya, two studies conducted in Nairobi reported an increase in the proportion of children attending Maternal Child Health (MCH) clinics in Nairobi who achieved minimum acceptable diet and dietary diversity following facility-based caregiver counselling using short video clips delivered over 3–5 months [16,17]. In Nigeria, nutrition counselling via short telephone text messages resulted in a 13% increase in the proportion of mothers who practised exclusive breastfeeding and a 15% increase in the proportion of children who achieved minimum meal frequency [18,19]. In China, mothers who received short telephone messages on breastfeeding reported a higher mean duration of breastfeeding of 11 weeks [20]. There is, however, limited data in the Kenyan setting on the role of mobile-health technology in enhanced caregiver counselling on locally available high-nutrient foods within the context of severe malnutrition. The objective of this study was to determine the effect of enhanced facility-based caregiver counselling on local high-nutrient foods, using a video with contextualized demonstrations of local high-nutrient foods (video-drama) and telephone text messages (SMS), on children’s median dietary diversity scores and mean rate of weight gain as well as on caregivers’ mean knowledge scores at 1, 6 and 12 weeks after enrolment. The hypothesis was that children in intervention study arms would have higher mean dietary diversity scores and mean rate of weight gain post-intervention while caregivers would demonstrate higher mean knowledge scores on local high-nutrient foods, compared to those receiving standard of care (SOC).

## Materials and methods

### Ethical considerations and trial registration

This study was approved by the University of Nairobi/Kenyatta National Hospital Ethics and Research Committee (UoN-KNH ERC) under Ref. KNH/ERC/A/234. It was registered with the Pan African Clinical Trials Registry (ID no. PACTR201905790643955) on 16^th^ May 2019. Written informed consent or consent by thumbprint sign, (approved by the UoN-KNH ERC), was obtained from parents/guardians before enrolment. The consenting process included an explanation to potential study participants of the likelihood, depending on their randomization study arm, of watching a nutrition education video-drama and receiving nutrition education messages through SMS. All participant data was stored in a password-protected computer with access only to the research team and was delinked of all participant identifiers before analysis. At study onset, the study PI trained research assistants in research ethics including confidentiality of participant data and study procedures.

### Study design and study site

This was a three-arm open-label, parallel intervention randomised controlled trial conducted at Mbagathi County Hospital (MCyH), a secondary-level referral hospital in Nairobi, Kenya. MCyH provides health services to children living in informal settlements within Nairobi with the estimated prevalence of severe wasting at 6.3% [21]). MCyH provides inpatient care to approximately 30 children with complicated SAM per month and in addition, has an outpatient nutrition (OPN) clinic that supplies RUTF to another 150 severely malnourished children on community follow-up. In the initial study period, we enrolled children at the time of discharge from the paediatric ward. However halfway through the study, following a study modification and protocol amendment occasioned by low numbers during the COVID-19 pandemic, we in addition enrolled children with SAM directly from the outpatient department.

### Inclusion and exclusion criteria

Child-caregiver pairs were consecutively enrolled, if children were 6–59 months old, had low weight-for-height (WHZ) score < -3SD or MUAC <11.5 cm, and had written informed caregiver consent. Children were excluded if they had underlying comorbidities (congenital heart disease, heart failure, pulmonary hypertension, cerebral palsy, malignancies, chromosomal abnormalities, e.g., Down syndrome, and any end-organ failure), that were likely to affect their dietary intake, if their caregivers did not own a mobile phone or were not planning to reside in Nairobi for the 12-week study period.

### Sample size and sampling technique

Using the formula for comparing means, we calculated the sample size required to identify a 20% difference in mean DDS of children comparing any of the intervention study arms to the SOC. We estimated a low baseline mean DDS of 2.58 with a standard deviation of 1.9, similar to what had previously been reported in India and Malawi [22,23]. We determined that 57 children per study arm would provide 80% power to detect this difference in DDS at 0.05 level of significance. The sample size was subsequently increased to 71 children per study arm to compensate for an anticipated 8% mortality rate and 15% loss-to-follow up giving a final sample size of 213 [3,24].

### Screening, enrolment, randomization, and blinding

Children presenting to the MCyH paediatric outpatient department and those admitted to the paediatric ward were screened for eligibility based on their age, presence of SAM based on WHZ score, and MUAC measurements, and presence of a parent/guardian at the time of screening. Participant enrolment began on 17-04-2019 and ended on 22-06-2021. Following informed consent, we used simple randomisation to allocate study participants at a ratio of 1:1:1 to the three study arms. Allocation was done by study research assistants using computer-generated random numbers, developed by the study statistician, which were placed in sequentially numbered externally labelled opaque sealed envelopes. Envelopes were only opened after participant enrolment. Based on available resources, only two research assistants were recruited for this study. The two research assistants who administered the intervention, obtained data on study outcomes. To ensure the objectivity of data obtained both research assistants were blinded to the study outcomes. We in addition used two different methods, the 24-hour recall method and 7-day food frequency questionnaire to obtain data for the primary objective of children’s dietary diversity scores. Due to the nature of the study intervention, which comprised a video-drama and telephone text messages on local high-nutrient foods from the seven WHO food groups, it was not possible to blind either the study Principal Investigator (PI), research assistants, or caregivers to the intervention. The study statistician was, however, blinded to the study intervention. The study PI was the only person aware of study outcomes, which were concealed from the research assistants and caregivers.

### Study intervention

#### Video-drama.

A video-drama providing health education messages on local high-nutrient foods was previously developed by the same research group in the local language, Kiswahili, as part of the parent research project (S1 Appendix). The nutrition education was delivered through a 10-minute dramatized video (video-drama). The drama act was done in one of the informal settlements within Nairobi by professional actors drawn locally from within Nairobi. The actors had previously acted in several local TV drama series and were sourced by the producer. The storyline revolved around domestic conflict within a family experiencing financial constraints caused by recurrent child illnesses due to malnutrition associated with low-nutrient home foods. Visual displays of locally available and affordable high-nutrient foods from the seven WHO food groups were included with their estimated costs and the recommended preparation methods as a way of ensuring optimal child nutrition.

Other key messages in the video included the importance of enriching children’s porridge through the addition of milk, oil and sugar, and the roles of animal meats and green leafy vegetables in bodybuilding and blood formation. Sugars, fats and oils were included as additional foods that should be provided in minimal amounts to increase the energy content of foods as well as to improve vitamin A absorption. Affordable foods were defined as foods with an estimated cost of Ksh 50 or less (0.5 USD) per piece/200ml portion depending on how these foods were sold at the local market. Food price estimates were determined at a market located within an informal settlement close to the study site. Pilot testing was done of the video-drama, using a sample of 20 caregivers whose children were admitted with SAM at MCyH during the time of video development. The pilot was aimed at assessing video acceptability in terms of the language used, time duration, and familiarity by caregivers with the foods presented.

#### Short text messages (SMS).

Twelve short text messages to be delivered via the short message system (SMS) were also developed by the study PI in Kiswahili, on local high-nutrient foods for children (S2 Appendix). The twelve telephone messages were selected based on the twelve-week study follow-up period and messages were sent out to participants in intervention arm B at a frequency of one message per week. A local SMS provider, mHealth Kenya, delivered nutrition text messages to caregivers using one-way bulk SMS. In the first 7 weeks text messages focused on individual food groups that caregivers should provide to children while subsequent messages focused on ways to enrich children’s porridge, the importance of fats and oils in vitamin A absorption, and the recommended meal frequency. The education component in the video-drama and SMS specifically focused on appropriate local high-nutrient foods for children with severe malnutrition and did not address RUTF consumption at home.

### Study arms

#### Standard of Care (SOC).

Caregivers received face-to-face counselling weekly, on local high-nutrient foods to provide to children, by the hospital nutritionists during follow-up visits to the OPN. Children were issued with a one-week supply of RUTF from the hospital nutrition clinic every week. Caregivers were advised to ensure that children completed the prescribed RUTF rations each day before offering other foods. **Intervention arm A:** Caregivers watched the video-drama at enrolment, 1, and 6 weeks in addition to receiving SOC. The video-drama was delivered to study participants individually in a private room within the Maternal Child Health (MCH) department at MCyH. The MCH department is located in a different compound within MCyH, approximately 100 metres from the hospital OPN clinic. To avoid contamination, follow-up study visits were scheduled in such a way that study participants came for their follow-up visits on different days, and when we had more than one participant on the same day, they were scheduled to come in at different times.

Participants were allowed to ask questions after watching the video-drama on aspects they may not have understood. Questions asked by caregivers included how early one can introduce foods like meat and eggs to children, preferred methods for cooking foods like eggs for young children, and whether foods like legumes and sardines could be added to children’s porridge. Other questions that were not content-related centered on reasons for delayed milestones and poor appetite in malnutrition, refusal by children to consume certain foods like green vegetables, causes and treatment of rickets. **Intervention arm B:** Caregivers received weekly telephone text messages (SMS) on local high-nutrient foods in addition to watching the video-drama at enrolment, 1 and 6 weeks, and receiving SOC.

### Study outcomes

The follow-up period for individual study participants from enrollment to the end of follow-up was 12 weeks. The primary study outcome was the difference in mean dietary diversity scores (DDS) of children at 1, 6, and 12 weeks. Secondary study outcomes were the rate of weight gain of children (in g/kg/day) and caregiver knowledge scores on local high-nutrient foods at 1, 6, and 12 weeks across study arms.

### Data collection procedures

To determine children’s dietary diversity scores, caregivers were interviewed on foods that children had consumed in the 24 hours preceding study visits. At enrolment, all caregivers were in addition provided with food diaries and requested to indicate the foods consumed by children during the seven days prior to study visits. To determine caregiver knowledge of local high-nutrient foods, we used six questions adapted from Module II of the Food and Agriculture Organization (FAO) guidelines on the assessment of caregiver knowledge of complementary feeding [25] (S3 Appendix). Questions assessed caregiver knowledge of the local high-nutrient foods contained in different food groups, ways to enrich children’s porridge, bodybuilding and iron-rich foods, cooking methods and foods to enhance vitamin-A absorption and the recommended minimum meal frequency for children. Children were weighed at each study visit and the mean rate of weight gain was calculated (in g/kg/day) at 1, 6, and 12 weeks.

Household food security was assessed for all study participants at enrolment using the Household Food Insecurity Assessment Scale (HFIAS) developed by Food and Nutrition Technical Assistance (FANTA) [26]. Household food security was considered a potential study confounder with regard to access by families to desired foods.

### Data analysis

Data was obtained using Open Data Kit (ODK) version 1.0 and analysed using R statistical software version 4.2.2. Inconsistencies and errors were identified and corrected. There were no protocol violations and analysis was done on an intent-to-treat basis. We did not do additional per protocol or sensitivity analysis.

#### Missing data.

Missing values for study outcomes were determined to be missing completely at random (MCAR), and not associated with the study intervention. For variables with low missingness (<10%), we used complete case analysis, as the variables are too few to impact analysis. Where there was low to moderate missingness (10%-30%), we employed median/mean imputation. Mean/median imputation was used to replace values for missing data based on the fact that compared to multiple imputation, it is simple to conduct and provides unbiased estimates when data is missing completely at random and study objectives do not include assessment for associations as was the case in our study.

Descriptive summary statistics were presented using frequencies and proportions for categorical and nominal variables, means and standard deviation or medians and interquartile range as appropriate for continuous variables. For the primary study outcome, we used the Kruskal Wallis test with post-hoc analysis using Dunn test with Bonferroni correction to compare differences in median DDS between study arms. Differences in the secondary study outcomes on children’s mean rate of weight gain and mean caregiver knowledge scores were compared using ANOVA with post-hoc analysis done using the Tukey HSD test. Differences were considered statistically significant if the p-value was < 0.05 for the initial analysis and <0.025 for the post-hoc analysis. Based on the study design where participants were randomly allocated to each of the three study arms and the balance in randomization that we achieved at baseline we did not control for any covariates in this study.

## Results

### Screening and enrolment and loss to follow-up

**Fig 1:**
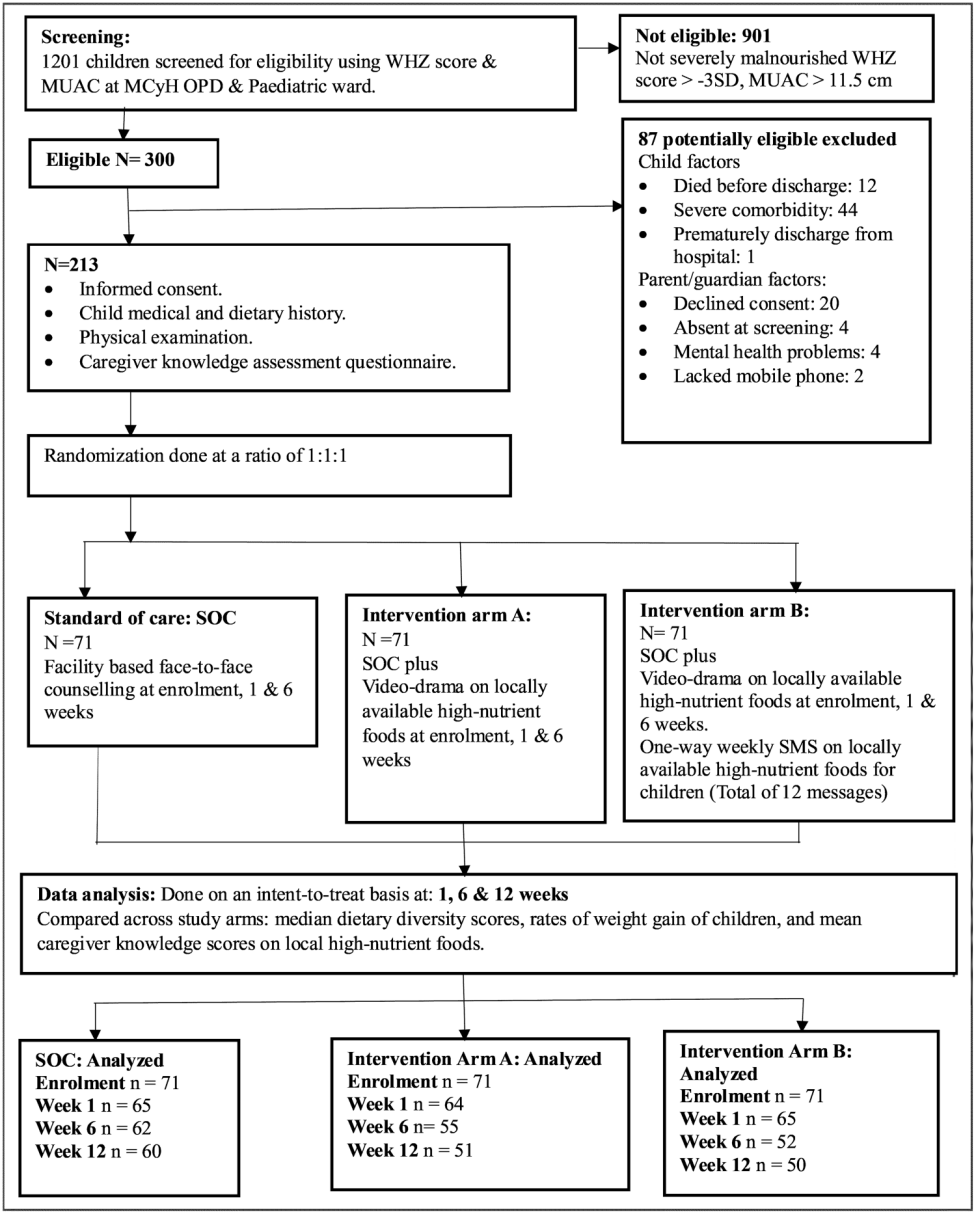
Flow chart showing screening, enrolment, randomization, study procedures, and follow-up.

Between 17^th^ April 2019 and 22^nd^ June 2021, 1201 children aged 6–59 months were screened for eligibility during hospital admission or at the time of outpatient clinic visit, of whom 300 (25.0%) had SAM and were potentially eligible (Fig 1). Twelve children died in hospital and among the 288 who survived and were discharged, 213 gave consent and were enrolled. Reasons for exclusion of 75 children were: lack of consent from the parent/guardian (20), absence of parent/guardian at the time of screening (4), and presence of an exclusion criterion (51). Exclusion criteria included: underlying comorbidities in 44 children (19 had cerebral palsy, 12 had heart disease, 11 had Down syndrome, 1 had pulmonary hypertension and 1 had liver disease). One child was prematurely discharged from the hospital (S1 Table). Post-enrolment 40 (18.8%) of the study participants were lost to follow-up due to various reasons. Eleven participants relocated out of the study area following the onset of the COVID pandemic, 12 failed to come for visits due to lockdown and financial reasons, 2 mothers were newly diagnosed with HIV during the study period and voluntarily withdrew from the study, and other 15 participants who failed to come for study visits could not be traced despite repeated attempts to contact them (S2 Table).

### Sociodemographic characteristics of study participants at enrolment

The median age of children at enrolment was 13 months (IQR 10,18), and there was a gender balance between males and females at 50% (Table 1). The median age for parents/guardians was 28 years (IQR 25, 32), all were female except for 1 male each in intervention arms A and B, and the median number of years in school for caregivers was 10 years (IQR 8, 12), corresponding to secondary level of education. There was a lower proportion of parents/guardians who were married in intervention Arm B (72%), compared to those in the SOC (82%) and intervention Arm A (78%), and most (66%) were homemakers. Fathers were identified as heads of household as well as main income earners in 70% of households. The median monthly income per household was estimated at 100 USD (IQR 62, 150), and approximately 20% of this income was spent on food at 21 USD (IQR 14, 28). The median number of people per household was 4 (IQR 3, 5), and the median number of children per household was 1 (IQR 1,2). Household food insecurity assessment score (HFIAS) was 18 out of a total score of 27, corresponding to moderate food insecurity. Less than 10% of households per study arm, were categorized as mildly food insecure, and 16% reported severe food insecurity. There was no difference in sociodemographic characteristics across study arms at enrolment.

**Table 1:** Sociodemographic characteristics of study participants at enrolment.

Participant characteristics		Standard of Care Median (IQR)/ Freq (%) (N = 71)	Intervention Arm A Median (IQR)/ Freq (%) (N = 71)	Intervention Arm B Median (IQR)/ Freq (%) (N = 71)
***Children’s characteristics***				
Age in months		11 (7, 17)	13 (10, 18)	12 (8, 16)
Female		39 (55)	36 (51)	37 (52)
***Parent/guardian characteristics***				
Caregiver age in years		28 (25, 32)	26 (23, 31)	29 (25, 33)
Number of years in school		10 (8, 12)	11 (8, 12)	10 (8, 12)
Sex-Female		71 (100)	70 (99)	70 (99)
Marital status- married		55 (78)	58 (82)	51 (72)
Occupation	Formal employment	6 (8.5)	7 (9.6)	6 (8.5)
	Casual	11 (15.5)	12 (16.9)	13 (18.3)
	Self-employed	9 (12.7)	9 (12.7)	8 (11.3)
	Homemakers	45 (63.4)	43 (60.6)	44 (62.0)
***Household characteristics***				
Head of household	Father	50 (70)	56 (79)	50 (70) ^a^
	Mother	16 (23)	12 (17)	12 (17)
	Other	5 (7)	3 (4)	9 (13)
Main income earner	Father	50 (70)	55 (78)	49 (69)
	Mother	13 (18)	11 (16)	12 (17)
	Other	8 (11)	5 (7.0)	10 (14) ^b^
Number of household members		4 (3, 5)	4 (3, 5)	5 (4,5)
Number of children per household		1 (1, 2)	1 (1, 2)	1 (1, 2)
Monthly income (USD)		100 (62, 150)	100 (60, 164)	100 (50-150)
Money spent on food (USD)		21 (14, 28)	21 (14, 29)	14 (14, 21)
Household food insecurity score (out of 27)		19 (16, 21)	18 (16, 20)	18 (15, 20)
Household food insecurity score (out of 27)	Mildly food insecure	3 (4.2)	4 (5.6)	7 (9.9)
	Moderately food insecure	54 (76.1)	55 (77.4)	58 (81.7)
	Severely food insecure	14 (19.7)	12 (16.9)	6 (8.5)

IQR = Interquartile range, USD = US dollar, ^a^ other included Grandmother (5), Aunt (3), and Grandfather (1), ^b^ other included Grandmother (5), Aunt (4), and Grandfather (1).

### Clinical and dietary characteristics of children at enrolment

The median weight of children at enrolment was similar across study arms at 6.3 kg (IQR 5.5, 6.8) while MUAC measurement was at 11.1 cm (IQR 10.5, 11.4) (Table 2). Less than 15% of children per study arm had oedema of malnutrition, and less than 10% had either TB or HIV. The majority of children (> 70%) were still breastfeeding at enrolment, and most (> 75%) had received RUTF from the health facility.

**Table 2:** Clinical and dietary characteristics of children at enrolment.

Characteristic	Standard of Care Freq (%)/ Median (IQR) (N = 71)	Intervention Arm A Freq (%)/ Median (IQR) (N = 71)	Intervention Arm B Freq (%)/ Median (IQR) (N = 71)
**Clinical characteristics**			
Weight (kg)	6.4 (5.3, 6.9)	6.4 (5.9, 7.1)	6.3 (5.5, 6.8)
MUAC (cm)	11.2 (10.5,11.4)	11.1 (10.5, 11.4)	11.1 (10.7,11.4)
Malnutrition-related oedema present	7 (9.9)	10 (14.1)	6 (8.5)
Living with HIV	0 (0.0)	5 (7.0)	3 (4.2)
Tuberculosis present	3 (4.2)	5 (7.0)	6 (8.5)
**Dietary characteristics**			
Still breastfeeding	56 (78.9)	53 (74.6)	53 (74.6)
Received RUTF	59 (83.1)	56 (78.9)	58 (81.7)

kg = kilograms, cm = centimetres, MUAC = Mid-Upper-Arm Circumference, HIV = Human Immunodeficiency Virus, RUTF = Ready-to-Use-Therapeutic-Food.

### Sociodemographic characteristics of study participants enrolled from the inpatient and outpatient departments

Out of 213 children enrolled in the study, 157 (73.7%) were from the inpatient department (IPD) and 56 (26.2%) were from the outpatient department (OPD). At baseline, the median number of years in school for caregivers of children enrolled from OPD was significantly higher at 12 months (IQR 8, 12) The children enrolled from OPD came from households with significantly higher median monthly income at 110 USD (IQR 55, 132), and with higher amount of income spent on food at 16 USD (IQR 11, 27). A significantly higher proportion of fathers were heads of household for children enrolled from the IPD and were main income earners at 122 (78%) and 121 (77%) respectively (S3 Table).

### Clinical and dietary characteristics of children enrolled from the inpatient and outpatient departments

Children from the OPD had a significantly higher median weight of 7.0 kg (IQR 6.7, 7.7) compared to those from IPD at enrolment (p-value <0.001). None of the children enrolled from the OPD had malnutrition-related oedema compared to 14.6% of those from the IPD,  6.4% of children from IPD had a diagnosis of TB compared to 7.1% of children from the OPD, and only 77.7% received RUTF at enrolment compared to 91.1% of those from the OPD (S4 Table).

### Comparison of children’s median dietary diversity scores (DDS) at 1, 6, and 12 weeks

Based on skewed data distribution for the primary study outcome on DDS that did not change with log transformation of the data, we reported median DDS and not mean DDS (S2 and S3 Figs).

#### 24-hour recall data.

**Fig 2:**
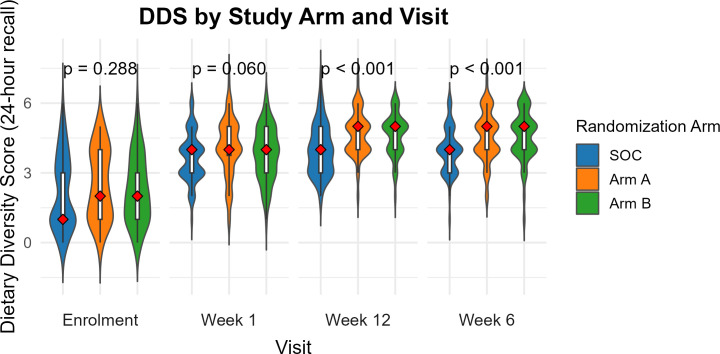
Comparison of children’s median dietary diversity scores using data from 24-hour recall interviews.

At baseline, children in all study arms had low median DDS at 1 (IQR 1, 3), 2 (IQR 1, 4), and 2 (IQR 1, 3) for the SOC and intervention arms A and B respectively (Fig 2). At 1 week, the median DDS improved to 4 out of 7 for all study arms. At 6 and 12 weeks there was significant difference in median DDS across study arms. Post-hoc analysis revealed significantly higher scores for intervention arms at 5 out of 7 compared to a score of 4 in the SOC. The median DDS for children in intervention arms A and B at 12 weeks was 5 (IQR 4, 5) compared to a score of 4 (IQR 3, 5) in the SOC (p-value < 0.001) (S5 Table).

#### 7-day food frequency data.

**Fig 3:**
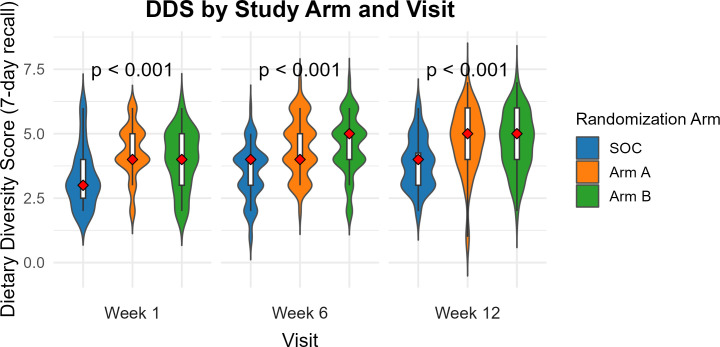
Comparison of children’s median dietary diversity scores using data from 7-day food frequency diary.

Results from the 7-day food frequency data were similar to what we reported using the 24-hour recall data (Fig 3). There was a significant difference in median DDS across the 3 study arms at 6 and 12 weeks. The median DDS was significantly higher at 4 and 5 (IQR 4, 5) out of 7 for children in the intervention arms A and B respectively at 6, and 12 weeks compared to those in the SOC whose median DDS was at 4 (IQR 3, 4) (p-value < 0.001). At 12 weeks children in intervention arms A and B had a higher median DDS of 5 (IQR 4, 6) compared to a median DDS of 4 (IQR 3, 4.5) in the SOC (p-value < 0.001) (S5 Table).

### Comparison of mean weights of children at 1, 6, and 12 weeks

**Fig 4:**
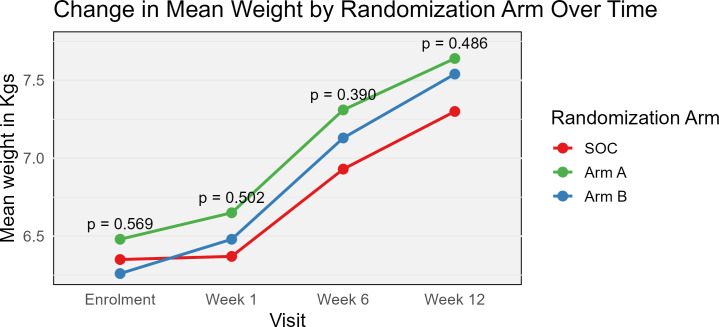
Comparison of mean weights of children.

Data was normally distributed for the two secondary study outcomes and for these, we reported the mean rate of weight gain and mean caregiver knowledge scores (S4 and S5 Figs). The mean weights of children gradually increased from 6.35 (± 1.38) kgs, 6.48 (± 1.16) kgs, and 6.26 (±1.23) kgs for SOC, intervention arms A and B respectively at enrolment to 7.30 (± 1.49) kgs, 7.64 (± 1.68) kgs and7.54 (± 1.56) kgs for SOC, intervention arms A and B at 12 weeks (Fig 4). Although we recorded higher mean weights for children in the intervention study arms compared to SOC, the difference in means was not statistically significant for any of the study visits.

### Comparison of children’s mean rate of weight gain in g/kg/day, at 1, 6, and 12 weeks

**Fig 5:**
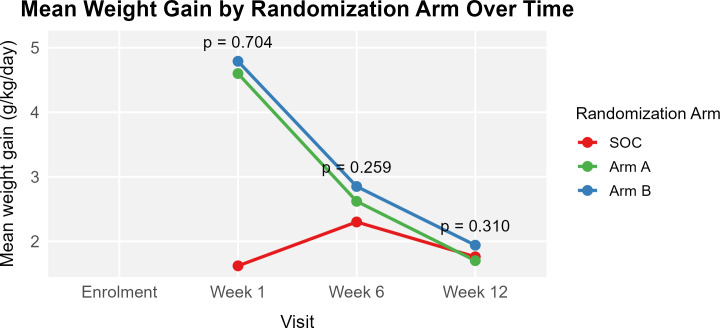
Comparison of the mean rate of weight gain of children across study arms.

The mean rate of weight gain remained low throughout the study period for children in the SOC (Fig 5). At 1 week it was at 1.62 (± 15.9) g/kg/day, at 6 weeks it was 2.30 (± 1.68) g/kg/day and at 12 weeks it was 1.76 (± 1.21) g/kg/day. Children in intervention study arms had a higher mean rate of weight gain at 1 week [4.60 (± 7.04) g/kg/day for arm A and 4.79 (± 7.75) g/kg/day for arm B]. This mean rate, however, gradually decreased to 1.70 (± 1.37) g/kg/day and 1.94 (± 1.13) g/kg/day at 12 weeks. The difference in mean rate of weight gain across study arms was again not statistically significant for any of the study visits.

### Comparison of mean caregiver knowledge scores on local high-nutrient foods at enrolment, 1, 6, and 12 weeks

**Fig 6:**
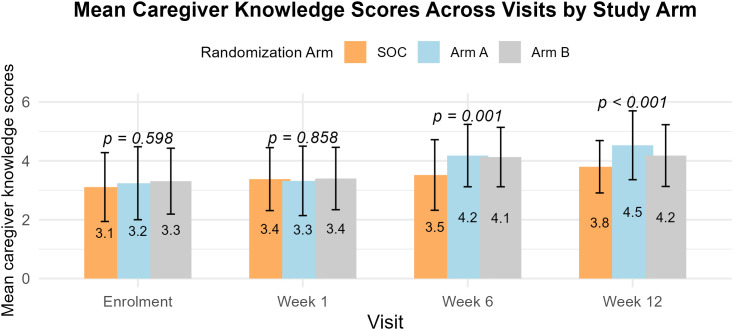
Comparison of mean caregiver knowledge scores on local high-nutrient foods for children.

At enrolment caregivers in all study arms had average knowledge scores on locally available high-nutrient foods with scores of 3.11 (± 1.17), 3.24 (±1.24), and 3.31 (±1.12) out of 6 for SOC and intervention arms A and B respectively (Fig 6). Mean knowledge scores remained average at 3 out of 6 at 1 week. At 6- and 12 weeks we noted significant difference in mean caregiver knowledge scores across the 3 study arms (p value 0.001 and <0.001 respectively). Post-hoc analysis revealed that caregivers in intervention arms A and B demonstrated significantly higher mean knowledge scores compared to those in SOC. At 12 weeks, caregivers in intervention arms A and B had mean knowledge scores of 4.53 (± 1.17) and 4.27 (± 1.04) compared to a mean score of 3.77 (± 0.91) in the SOC (p values <0.001and 0.036 respectively) (S5 Table).

### Caregiver performance on specific knowledge components

At the end of the 12-week study period, a higher proportion of caregivers in intervention study arms correctly identified foods from 4 different food groups as essential for a nutritionally adequate diet (68.6% and 52.0% for intervention arms A and B respectively, and 36.6% in SOC) (Table 3). More caregivers in intervention study arms also knew how to enrich children’s porridge at 68.6% and 74.5% for intervention arms A and B compared to 60.0% in the SOC. More caregivers in intervention study arms identified animal meats as body-building foods (62.8% and 56.0% for intervention arms A and B and 43.8% for SOC). A similar proportion of caregivers across the 3 study arms had correct knowledge of the recommended minimum meal frequency for children at 12 weeks (68.3%, 68.0%, and 70.0% for SOC, intervention arms A and B respectively).

**Table 3:** Caregiver performance on individual knowledge components in local high-nutrient foods.

Knowledge component	Study Visit	Standard of care n (%)	Intervention Arm A n (%)	Intervention Arm B n (%)
Identification of foods from different food groups	Enrolment	16 (22.5)	16 (22.5)	23 (36.6)
	Week 1	19 (29.2)	20 (32.3)	21 (30.8)
	Week 6	16 (25.8)	28 (50.9)	21 (40.4)
	Week 12	22 (36.6)	35 (68.6)	26 (52.0)
Importance of oil in vitamin A absorption	Enrolment	45 (68.2)	54 (76.1)	46 (67.6)
	Week 1	44 (67.7)	44 (71.0)	45 (69.7)
	Week 6	50 (80.7)	38 (69.1)	41 (78.9)
	Week 12	52 (86.7)	40 (78.4)	41 (82.0)
Methods to enrich children’s porridge	Enrolment	44 (66.8)	39 (60.9)	44 (61.9)
	Week 1	34 (52.3)	30 (46.9)	38 (83.1)
	Week 6	41 (66.1)	24 (42.8)	29(56.9)
	Week 12	36 (60.0)	35 (68.6)	38 (74.5)
Role of animal meats as body-building foods	Enrolment	35 (49.3)	33 (46.5)	39 (54.9)
	Week 1	34 (52.3)	33 (53.2)	32 (49.2)
	Week 6	35 (56.5)	30 (54.6)	30 (57.7)
	Week 12	29 (48.3)	32 (62.8)	28 (56.0)
Role of dark green vegetables as iron-rich foods for blood formation	Enrolment	26 (36.6)	22 (31.0)	26 (36.6)
	Week 1	28 (43.1)	22 (35.5)	27 (41.5)
	Week 6	24 (38.7)	26 (47.3)	21 (40.4)
	Week 12	33 (55.0)	21 (41.2)	19 (38.0)
Recommended meal frequency for children	Enrolment	35 (49.3)	43 (60.6)	36 (50.7)
	Week 1	40 (61.5)	38 (61.3)	42 (64.6)
	Week 6	38 (61.3)	37 (67.3)	31 (59.6)
	Week 12	41 (68.3)	35 (68.6)	35 (70.0)

There was, however, a lower proportion of caregivers in intervention study arms compared to those in SOC who mentioned cooking with oil to increase vitamin A absorption in foods (86.5%, 78.4%, and 82.0% for SOC, intervention arms A and B respectively). Fewer caregivers in intervention study arms identified dark green vegetables as iron-rich foods important for blood formation post-study intervention (41.2% and 38.0% for intervention arms A and B respectively compared to 55.0% in the SOC).

### Mortality among children during the study period

We reported 12 deaths among children during the study period giving an overall mortality rate of 5.6%. There were 3 (4.2%) deaths in the SOC, 3 (4.2%) in intervention Arm A, and 6 (8.5%) in intervention Arm B. The majority of deaths occurred during the first 6 weeks at 2 out 3 deaths for both the SOC and intervention arm A and 5 out 6 deaths for intervention arm B. Deaths were not associated with the study intervention (p-value > 0.05 comparing each intervention arm to SOC) (S6 Fig).

### Hospital admissions among children during the study period

There were 23 (10.7%) hospital admissions among children during the study period. Three (4.2%) admissions occurred in the SOC at 5, 6, and 10 weeks. Six (8.5%) admissions occurred in intervention arm A, 2 in the first 3 weeks and 1 at 6 weeks. There were 14 (19.7%) admissions in intervention arm B and the majority (10) occurred in the first 3 weeks. Severe pneumonia and acute diarrhoeal disease were the main reasons for admission. Admissions were not related to the study intervention (p-value > 0.05 comparing the intervention study arms to SOC) (S7 Fig).

## Discussion

Our study findings highlight the effect of enhanced caregiver counselling on locally available high-nutrient foods for severely malnourished children. We found that a video-drama significantly improved children’s dietary diversity scores and caregiver knowledge of these foods beyond routine standard-of-care counselling. The rate of weight gain among children in intervention study arms did not significantly differ between arms and there was no additional benefit of weekly SMS messages on local high-nutrient foods to caregivers.

While all children in this study had severe malnutrition at enrolment, only 8 out of 10 received RUTF from the outpatient nutrition clinic. Failure by children to access RUTF from nutrition clinics, as well as poor adherence reported in studies, even when RUTF is available, highlights the fact that children with SAM often have to rely on local foods to achieve weight gain and recovery while at home. It is, therefore, imperative that caregivers are equipped with adequate knowledge of locally available high-nutrient foods and that children’s diets contain these foods to fill the nutrient gap not met by RUTF.

The key finding in our study was the significant effect that the video-drama had on the median dietary diversity scores of children. By the end of the study period, children in the intervention study arms achieved a median dietary score of 5 out of 7, while that of children in the SOC remained at 4 out of 7. While other nutrition counselling interventions in Kenya and Peru have demonstrated similar improvements in children’s diets, these interventions were costly as they were delivered over a longer period of 12–18 months and delivered counselling messages through home visits by health workers with hands-on cooking demonstrations [27,28]. The use of video-drama in the current study provides a less costly approach to enhanced counselling on local high-nutrient foods in the absence of additional health personnel to conduct home visits. Failure to demonstrate additional benefits with SMS may have resulted from the fact that caregivers received telephone messages after having watched the video-drama. Unlike in the video-drama, which was presented in the form of a drama with visual displays of relevant foods, telephone messages were presented in text form, and caregivers may not have been as motivated to read the messages or to retain the information received through text messages.

At enrolment, children had a low median dietary diversity score of less than 4 out of 7. This low score mirrors previous findings from Kenya and India, where children on complementary feeding demonstrated low scores of 3.40 and 2.58, respectively [27,22]. More recently, a national survey that enrolled over 70,000 children in India found that approximately 54% of children in India consumed foods from less than 2 food groups daily [29]. Earlier studies in Kenya and Tanzania described children’s diets as mainly cereal-based and often lacking in proteins, vitamin A-rich fruits, and green vegetables [30,31]. Our findings on low dietary diversity highlight the need for effective interventions to improve diet quality for children at home.

Post-intervention, caregivers in the intervention study arms had significantly higher mean knowledge scores compared to those in the SOC. They also correctly identified foods from different food groups and understood the role of animal meats as body-building foods and methods to enrich porridge. These results support our study hypothesis that a contextualized video-drama on local high-nutrient foods, in addition to SOC, does improve caregiver knowledge of these foods and subsequently leads to improved dietary diversity for malnourished children. From our study findings, we extrapolated that the intervention improved caregiver knowledge of local high-nutrient foods, which then impacted caregiver-child feeding practices and improved children’s dietary diversity scores. Inconsistent results on some of the knowledge components may have resulted from the fact that this was a secondary objective, and we may not have had adequate power to examine for outcomes on individual knowledge components.

Weight gain is a product of multiple factors beyond dietary diversity including the presence or absence of illness, consumption of the right amount of food consumed, and the correct feeding frequency. Inadequacies in any of these factors could explain our failure to demonstrate significant weight gain among children in intervention study arms despite having demonstrated significant improvements in caregiver knowledge and children’s dietary diversity scores. We, however, noted that the rate of weight gain across all three study arms remained low at less than 5 g/kg/day for all study visits.

Most of our study participants, though drawn from informal settlements within Nairobi, reported moderate household food insecurity. Although caregivers reported challenges accessing the types of food they desired, they did not report a complete lack of food within households. Previous studies conducted within informal settlements have reported similar findings, where most households in this setting tend to experience some level of food insecurity, but often not the most severe form [32,33]. In a study from South Africa, out of 160 households assessed, 59% experienced mild to moderate food insecurity, while 8% were food-secure [32]. In another study from Kenya, 28% of 6,500 households sampled from two slum areas in Nairobi were categorized as food secure, while 50% had moderate food insecurity [33]. With adequate counselling, therefore, caregivers in this setting would be able to channel limited finances towards the purchase of affordable high-nutrient foods.

The mortality rate of 5.6% that we reported among children in our study was lower than the 8.7% reported in Bangladesh and 10% reported in Malawi among children with severe malnutrition in the first 3 months post-hospital discharge [3,4]. Of note is the fact that in the study in Malawi, 45% of the children enrolled were living with HIV and this group contributed 64% of the deaths reported [4]. The study population in the study in Bangladesh was somewhat younger compared to ours with a median age of 10 months. The median age of children in our study was 12 months and less than 10% were HIV positive. Our findings on pneumonia and diarrhoea as the main conditions associated with mortality were consistent with findings in Bangladesh and Malawi [3,4].

### Study strengths and limitations

Our main study strength was the randomized controlled design which allowed for control of known and unknown study confounders. Another study strength was the innovativeness of the intervention where we used a drama-act to convey key messages on appropriate local high-nutrient foods for children, a creative approach that captured the attention of the caregivers and enabled them to identify with the study setting.

Our study had several limitations. The primary outcome on children’s dietary diversity scores had missing data for 29% of the study participants who either died or were lost to follow-up during the study period. Part of this study was conducted during the COVID-19 pandemic resulting in a higher-than-expected loss to follow-up among study participants, many of whom failed to show up for study visits due to relocation to their rural homes or fear of contracting COVID-19. We in addition relied on caregivers’ reports on children’s dietary intake using the 24-hour recall dietary intake with the likelihood for desirability bias. To mitigate this, we in addition obtained data using the 7-day food diary documented daily by caregivers to allow for comparability of responses. Participants may however have still modified responses. The third limitation was a lack of direct observations of food intake by children as well as measurement of quantities of foods consumed, which would have been the most reliable way to document dietary intake. This was not possible due to financial and time constraints. Our study also took longer than anticipated due to multiple health worker strikes during the study period.

### Generalisability of study results

This study demonstrates improvement in child dietary diversity scores and caregiver knowledge of local high-nutrient foods among child-caregiver dyads at a secondary-level facility within a low socio-economic urban setting. Our results are likely generalisable to children with severe malnutrition receiving care in similar settings and their caregivers. The generalisability of results to children and caregivers in higher socioeconomic and rural settings requires further study.

## Conclusions and recommendations

In conclusion, the use of contextualized video-drama, to enhance SOC counselling to caregivers on local high-nutrient foods for children with severe malnutrition, was effective in improving children’s median dietary diversity scores and mean caregiver knowledge scores. We found weight increases but no significant difference in weight gain in the intervention arm. Longer term follow-up may be needed to ascertain the long-term impact of improved dietary diversity. Video-drama is a feasible cost-effective intervention that can be easily scaled up in similar settings to enhance facility-based caregiver counselling on high-nutrient foods to provide to children.

## Supporting information

S1 Fig The seven WHO food groups for assessing dietary diversity in infants and children. (TIFF)

S2 Fig Data distribution curves: Log transformed data on children’s dietary scores using 24-hour recall data. (DOCX)

S3 Fig Data distribution curves: Log transformed data on children’s dietary scores using 7-day food data. (DOCX)

S4 Fig Data distribution curves: Log transformed data on mean weights of children. (DOCX)

S5 Fig Data distribution curves: Log transformed data on mean caregiver knowledge scores. (DOCX)

S6 Fig Kaplan Meier curve: Mortality among children during the study period. (DOCX)

S7 Fig Kaplan Meier curve: Hospital admissions among children during the study period. (DOCX)

S1 Appendix Video-drama on local high-nutrient foods for children. (MP4)

S2 Appendix Telephone text messages on local high-nutrient foods for children (English version). (DOCX)

S3 Appendix Questions assessing caregivers’ knowledge of local high-nutrient foods. (DOCX)

S1 Table Reasons for exclusion of potential study participants. (DOCX)

S2 Table Loss to follow-up (LFTU) among study participants. (DOCX)

S3 Table Sociodemographic characteristics of study participants enrolled from the Inpatient and Outpatient departments. (DOCX)

S4 Table Clinical and dietary characteristics of children enrolled from the Inpatient and Outpatient departments. (DOCX)

S5 Table Post-hoc results for study visits with significant tests on Kruskal-Wallis and ANOVA tests. (DOCX)

S1 Data Enrolment data. (XLSX)

S2 Data Study follow-up data. (XLSX)

S3 Data 24-hour recall dietary data. (XLSX)

S4 Data 7-day food frequency data. (XLSX)

S5 Data Caregiver knowledge scores. (XLSX)

S1 File CONSORT Checklist. (PDF)
