# Supplementary figures and images for: Effect of diet video-drama and telephone messages on improving parental knowledge and diet diversity of malnourished children in Kenya: A randomised controlled trial
Source: PLOS Glob Public Health. 2025 Jul 9;5(7):e0004818. doi: 10.1371/journal.pgph.0004818 (PMC12240368; doi:10.1371/journal.pgph.0004818)

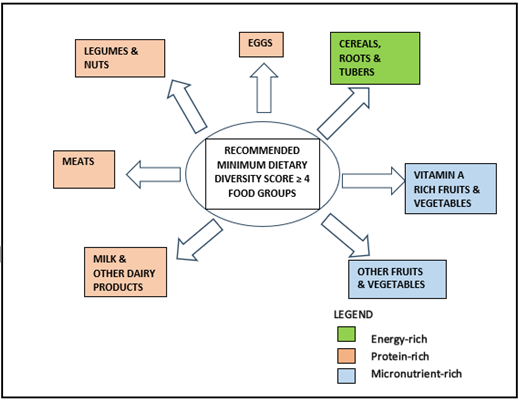

Supplement: S1 Fig — (TIFF) [file pgph.0004818.s001.tiff]

**S4 Fig. Data distribution curve: Mean weights of children at enrolment, 1-, 6- and 12 weeks**

**
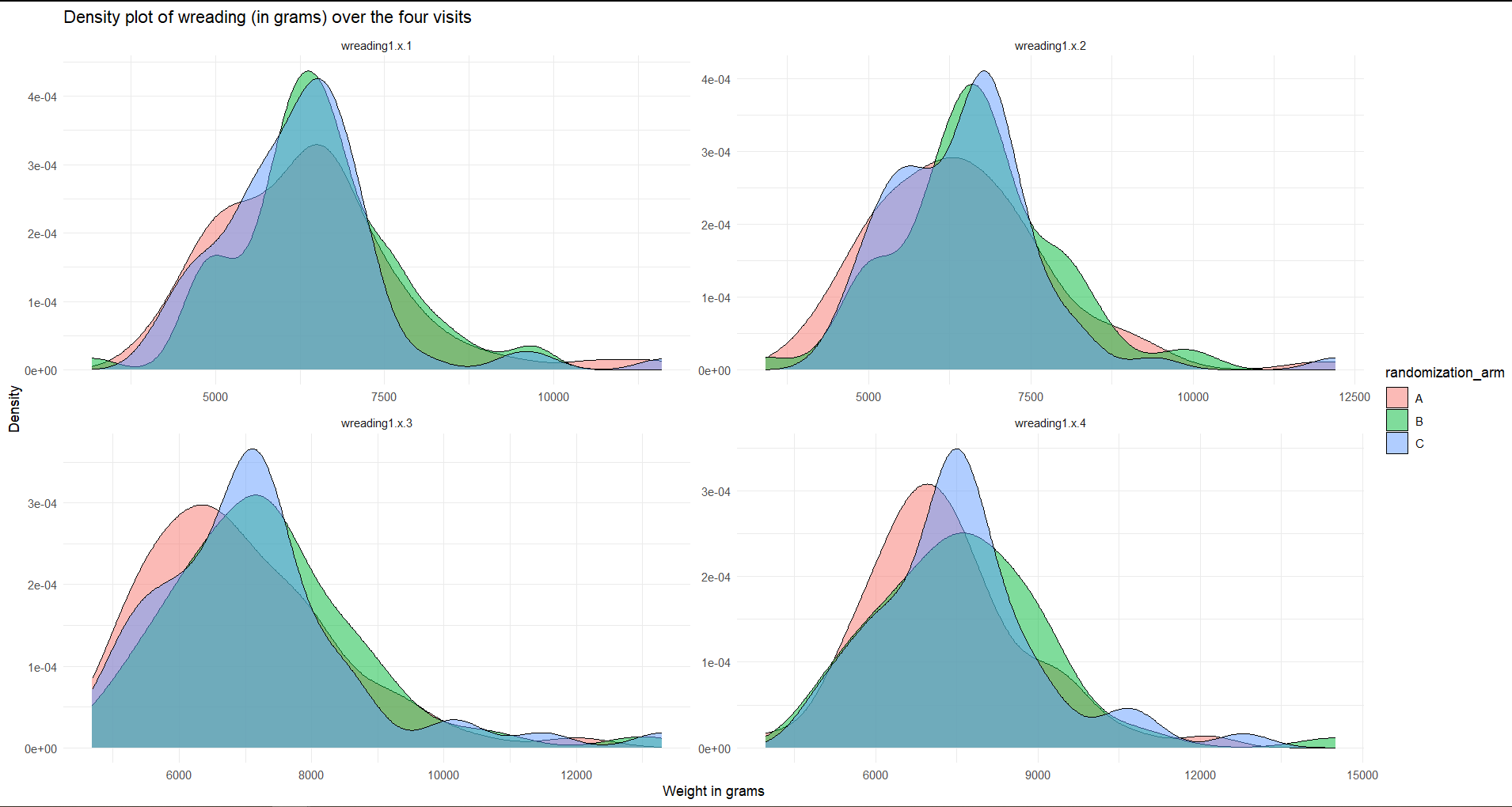
**

Supplement: S4 Fig — (DOCX) [file pgph.0004818.s004.docx]

**S6 Fig. Mortality among study participants during the 12-week study period**


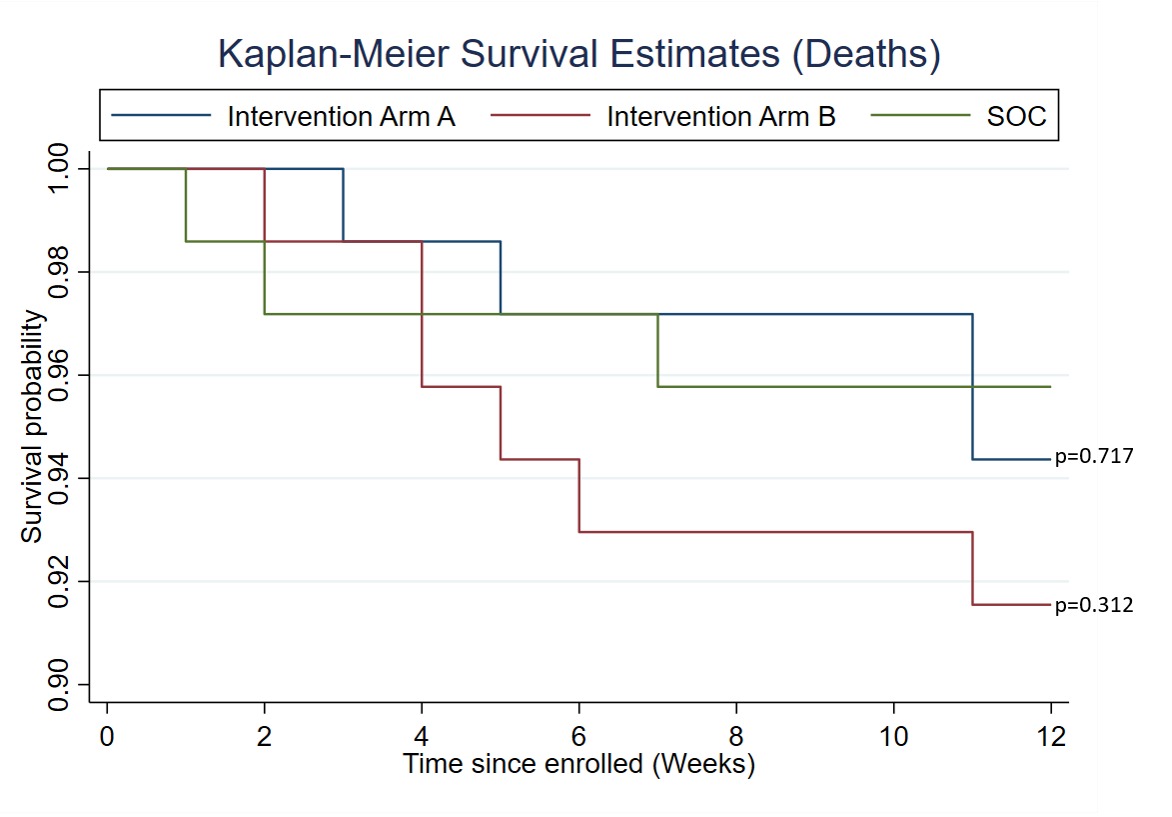

Supplement: S6 Fig — (DOCX) [file pgph.0004818.s006.docx]

**S7 Fig. Hospital admissions among study participants during the 12-week study period**


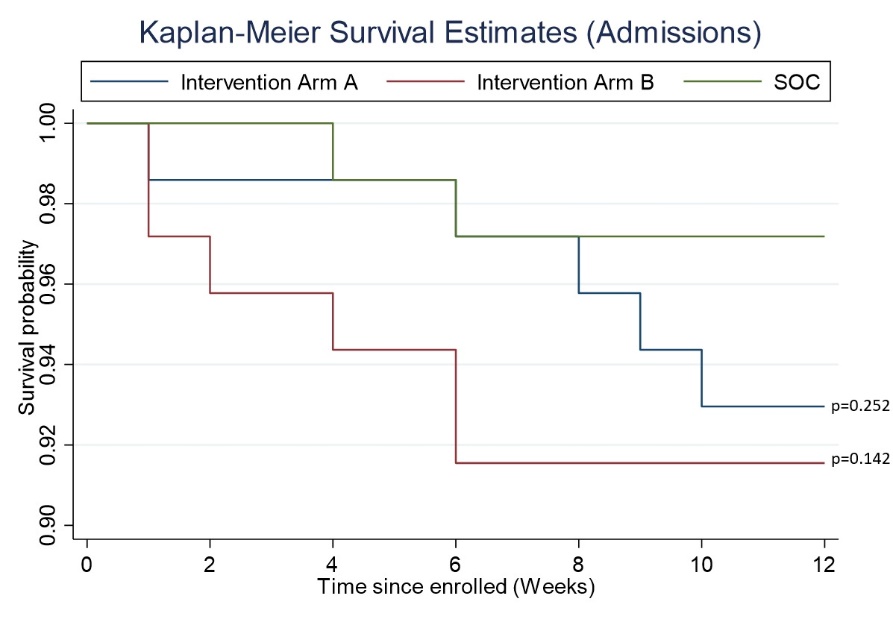

Supplement: S7 Fig — (DOCX) [file pgph.0004818.s007.docx]
