# Supplementary material for: Effect of diet video-drama and telephone messages on improving parental knowledge and diet diversity of malnourished children in Kenya: A randomised controlled trial
Source: PLOS Glob Public Health. 2025 Jul 9;5(7):e0004818. doi: 10.1371/journal.pgph.0004818 (PMC12240368; doi:10.1371/journal.pgph.0004818)

**S2 Fig. Log-transformed data distribution: Children’s dietary diversity scores using 24-hour recall data at enrolment, 1-, 6- and 12 weeks.**


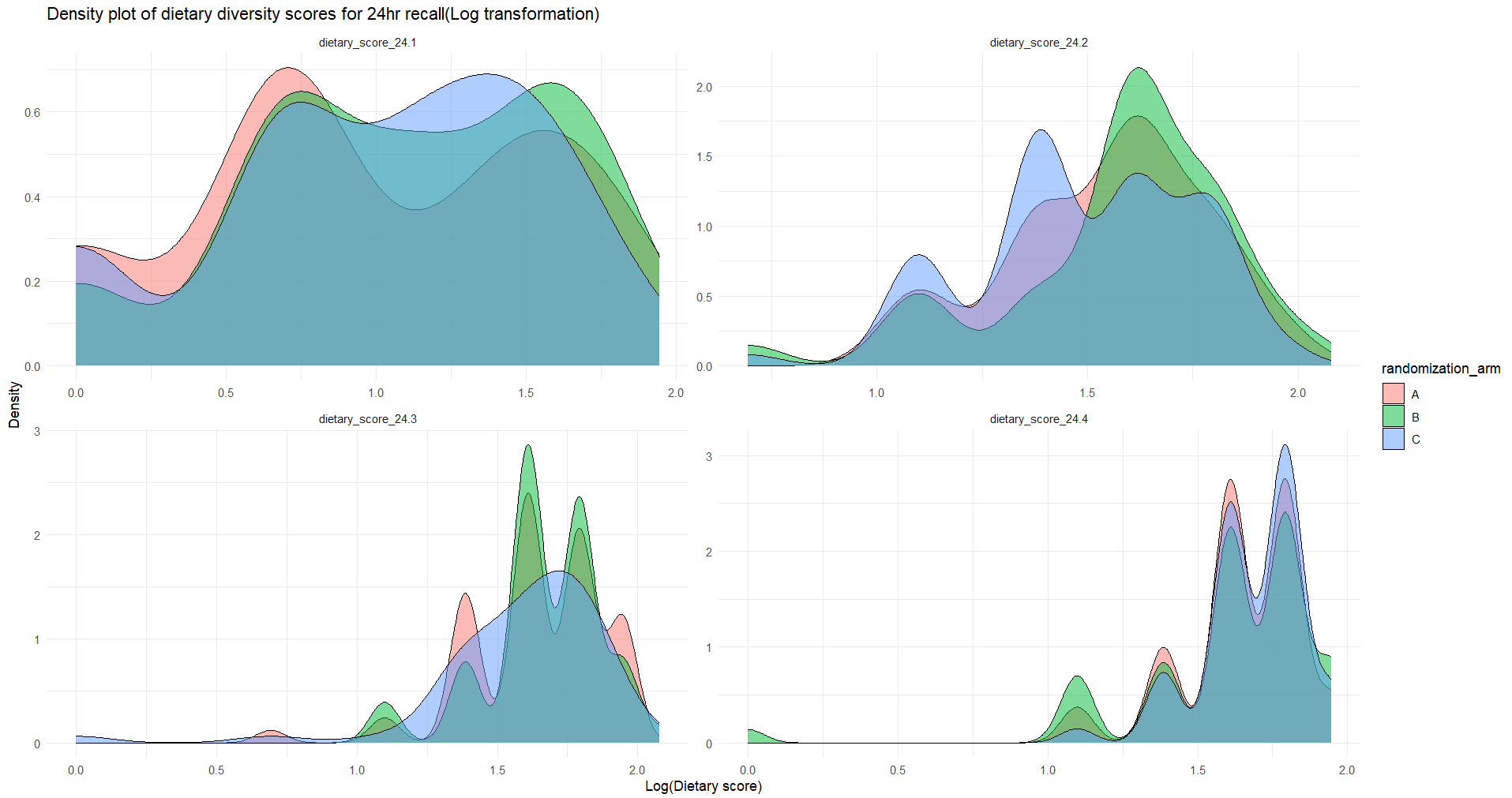

Supplement: S2 Fig — (DOCX) [file pgph.0004818.s002.docx]
