# Supplementary material for: Effect of diet video-drama and telephone messages on improving parental knowledge and diet diversity of malnourished children in Kenya: A randomised controlled trial
Source: PLOS Glob Public Health. 2025 Jul 9;5(7):e0004818. doi: 10.1371/journal.pgph.0004818 (PMC12240368; doi:10.1371/journal.pgph.0004818)
Supplement: S2 Appendix — (DOCX) [file pgph.0004818.s009.docx]

**S2 Appendix. Telephone text messages on local high-nutrient foods for children**

**(English version):**

1. Continue breastfeeding your child until the age of 24 months. Provide cow’s milk to your child from the age of 1 year as it contains calcium for strong bones (158 characters)

2. Remember to feed your child body-building foods like sardines, chicken wings so that they gain weight quickly (110 characters)

3. Remember to provide foods that are rich in vitamin A like pawpaw, mangoes and pumpkin to boost your child’s immunity and eyesight (130 characters)

4. Remember to provide eggs to your child often (45 characters)

5. Provide energy-giving foods like ugali, rice, potatoes and wimbi or maize porridge regularly to your child (107 characters)

6. 6. Provide body-building foods like beans, peas, lentils (ndengu) to your child regularly (87 characters)

7. Ensure that your child eats green vegetables like kales, spinach, terere and managu daily as they contain iron and are good for blood formation (144 characters)

8. Ensure your child eats foods from 4 or more food groups daily to ensure they receive adequate nutrients (104 characters)

9. Ensure that your child’s porridge is thick and does not easily pour from the spoon (83 characters)

10. Add a little oil, sugar and milk to your child’s porridge to increase the nutrient content (91 characters)

11. Add a little oil to your child’s food when cooking to improve vitamin A absorption (83 characters)

12. Ensure that you feed your child at least 5 times in a day to ensure that they receive adequate nutrients (104 characters)
