# Supplementary material for: Effect of diet video-drama and telephone messages on improving parental knowledge and diet diversity of malnourished children in Kenya: A randomised controlled trial
Source: PLOS Glob Public Health. 2025 Jul 9;5(7):e0004818. doi: 10.1371/journal.pgph.0004818 (PMC12240368; doi:10.1371/journal.pgph.0004818)
Supplement: S3 Appendix — (DOCX) [file pgph.0004818.s010.docx]

**S3 Appendix. Questions assessing caregivers’ knowledge of local high-nutrient foods**

1. Please name 4 types of foods, (excluding oils and sugars) that you would provide in your child’s meal to ensure that it has adequate nutrients for growth.

i) _____________________

ii) _____________________

iii)_____________________

iv) _____________________

2. Name one method you would use when cooking to improve vitamin A absorption in your child’s food.

__________________________________________________________________________

3. Name ways that you would use to improve the nutrient content of porridge that you have prepared for your child.

____________________________

____________________________

____________________________

____________________________

4. Name two foods that are important for bodybuilding.

____________________________

____________________________

5. Name one iron-containing food that is important for increasing blood levels in children.

___________________________

6. What is the recommended number of times that you should feed your child in a day to ensure they receive an adequate amount of nutrients?

_____________________times per day.
