# Supplementary material for: Effect of diet video-drama and telephone messages on improving parental knowledge and diet diversity of malnourished children in Kenya: A randomised controlled trial
Source: PLOS Glob Public Health. 2025 Jul 9;5(7):e0004818. doi: 10.1371/journal.pgph.0004818 (PMC12240368; doi:10.1371/journal.pgph.0004818)
Supplement: S1 Table — (DOCX) [file pgph.0004818.s011.docx]

S1 Table: Reasons for exclusion of potential study participants

| **Reasons for exclusion** | **Number excluded.**  **Frequency (%)**  **N = 75** |
| --- | --- |
| Child factors |  |
| Underlying comorbidity   - Cerebral palsy - Congenital Heart disease - Down syndrome - Pulmonary hypertension - Liver disease   Discharged against medical advice | 19 (25.3)  12 (16.0)  11(14.7)  1(1.3)  1(1.3)  1(1.3) |
| Parent/guardian factors |  |
| Parent/guardian absent during screening  Mental health problems in the mother  Lack of a mobile phone  Declined consent for participation | 4(5.3)  4 (5.3)  2 (2.7)  20 (26.7) |
