# Supplementary material for: Effect of diet video-drama and telephone messages on improving parental knowledge and diet diversity of malnourished children in Kenya: A randomised controlled trial
Source: PLOS Glob Public Health. 2025 Jul 9;5(7):e0004818. doi: 10.1371/journal.pgph.0004818 (PMC12240368; doi:10.1371/journal.pgph.0004818)
Supplement: S2 Table — (DOCX) [file pgph.0004818.s012.docx]

**S2 Table: Loss to follow-up (LFTU) among study participants**

| **Reasons for LFTU** | **STUDY ARM** | | | | | | **TOTAL** |
| --- | --- | --- | --- | --- | --- | --- | --- |
|  | **SOC** | | **ARM A** | | **ARM B** | |  |
|  | **No.** | **Time of LFTU** | **No.** | **Time of LFTU** | **No.** | **Time of LFTU** |  |
| Relocated outside of Nairobi | 2 | Week 1- 1  Week 6 - 1 | 3 | Week 1 - 1  Week 6 - 1  Week 12 - 1 | 6 | Week 1 - 2  Week 6 - 3  Week 12- 1 | 11 |
| Missed study visits and could not be traced despite repeated attempts | 3 | Week 1- 2  Week 12- 1 | 7 | Week 1 - 2  Week 6 - 4  Week 12 - 1 | 5 | Week 1- 1  Week 6 - 3  Week 12 - 1 | 15 |
| Missed study visit due to COVID lockdown/financial reasons | 3 | Week 1 - 1  Week 6 - 1  Eek 12 - 1 | 6 | Week 1 - 5  Week 6 - 3 | 3 | Week 1 – 1  Week 6 - 2 | 12 |
| Mother newly diagnosed with HIV during the study period and withdrew from study | 0 | N/A | 1 | Week 12 - 1 | 1 | Week 6 - 1 | 2 |
| **TOTAL** | **8** | | **17** | | **15** | | **40** |
