# Supplementary material for: Effect of diet video-drama and telephone messages on improving parental knowledge and diet diversity of malnourished children in Kenya: A randomised controlled trial
Source: PLOS Glob Public Health. 2025 Jul 9;5(7):e0004818. doi: 10.1371/journal.pgph.0004818 (PMC12240368; doi:10.1371/journal.pgph.0004818)
Supplement: S3 Table — (DOCX) [file pgph.0004818.s013.docx]

**S3 Table: Baseline characteristics of participants enrolled from the Outpatient (OPD) and Inpatient (IPD) Departments**

| **Participant characteristics** | | **Inpatient Department**  Median (IQR)/  Freq (%)  **(N = 157)** | **Outpatient Department**  Median (IQR)/  Freq (%)  **(N = 56)** | **p-value** |
| --- | --- | --- | --- | --- |
| ***Children’s characteristics*** | | | | |
| Age in months | | 13 (9, 17) | 11 (8, 17) | 0.27 |
| Female | | 86 (55) | 26 (46) | 0.28 |
| ***Parent/guardian characteristics*** | | | | |
| Caregiver age in years | | 28.0 (25, 32) | 29.0 (25, 33) | 0.977 |
| Number of years in school | | 10 (8, 12) | 12 (8,12) | 0.006 |
| Sex-Female | | 155 (98.7) | 56 (100) | 1.00 |
| Marital status- married | | 127 (81) | 37 (66) | 0.092 |
| Occupation | Formal employment | 15 (10) | 4 (7) | 0.800 |
|  | Casual | 18 (12) | 8 (14) |  |
|  | Self-employed | 25 (16) | 11 (20) |  |
|  | Homemakers | 99 (63) | 33 (59) |  |
| ***Household characteristics*** | | | | |
| Head of household | Father | 122 (78) | 34 (61) | 0.013 |
|  | Mother | 27 (17) | 13 (23) |  |
|  | Other | 8 (5) | 9 (16) |  |
| Main income earner | Father | 121 (77) | 33 (59) | 0.016 |
|  | Mother | 24 (15) | 12 (21) |  |
|  | Other | 12 (8) | 11 (20) |  |
| Number of household members | | 4 (3, 5) | 4 (3, 5) | 0.540 |
| Number of children per household | | 1 (1, 2) | 1 (1, 2) | 0.440 |
| Monthly income (USD) | | 76 (39, 116) | 110 (55, 132) | 0.020 |
| Money spent on food (USD) | | 14 (11, 16) | 16 (11, 27) | 0.016 |
| Household food insecurity score (out of 27) | | 18 (17, 20) | 19 (17, 21) | 0.200 |

IQR = Interquartile range, USD = US dollar, ^a^ other included Grandmother (5), Aunt (3), and Grandfather (1), ^b^ other included Grandmother (5), Aunt (4), and Grandfather (1).
