# Supplementary material for: Effect of diet video-drama and telephone messages on improving parental knowledge and diet diversity of malnourished children in Kenya: A randomised controlled trial
Source: PLOS Glob Public Health. 2025 Jul 9;5(7):e0004818. doi: 10.1371/journal.pgph.0004818 (PMC12240368; doi:10.1371/journal.pgph.0004818)
Supplement: S4 Table — (DOCX) [file pgph.0004818.s014.docx]

**S4 Table: Clinical and dietary characteristics of children enrolled from the Outpatient (OPD) and Inpatient (IPD) departments**

| **Characteristic** | **Inpatient Department**  Freq (%)/  Median (IQR)  **(N = 157)** | **Outpatient Department**  Freq (%)/  Median (IQR)  **(N = 56)** | p-value |
| --- | --- | --- | --- |
| **Clinical characteristics** | | | |
| Weight (kg) | 6.0 (5.2, 6.5) | 7.0 (6.7, 7.6) | <0.001 |
| MUAC (cm) | 11.2 (10.5, 11.4) | 11.1 (10.8, 11.4) | 0.690 |
| Malnutrition-related oedema present | 23 (14.6) | 0 (0) | 0.002 |
| Living with HIV | 7 (4.5) | 1 (1.8) | 0.200 |
| Tuberculosis present | 10 (6.4) | 4 (7.1) | < 0.001 |
| **Dietary characteristics** | | | |
| Still breastfeeding | 117 (74.5) | 45 (80.4) | 0.380 |
| Received RUTF | 122 (77.7) | 51 (91.1) | 0.028 |

IQR = Interquartile range, USD = US dollar, ^a^ other included Grandmother (5), Aunt (3), and Grandfather (1), ^b^ other included Grandmother (5), Aunt (4), and Grandfather (1).
