# Supplementary material for: Effect of diet video-drama and telephone messages on improving parental knowledge and diet diversity of malnourished children in Kenya: A randomised controlled trial
Source: PLOS Glob Public Health. 2025 Jul 9;5(7):e0004818. doi: 10.1371/journal.pgph.0004818 (PMC12240368; doi:10.1371/journal.pgph.0004818)
Supplement: S5 Table — (DOCX) [file pgph.0004818.s015.docx]

**S5 Table: Post-hoc analysis results for study visits with significant tests on Kruskal-Wallis and ANOVA**

| **Visits with significant results on the Kruskal-Wallis test**  **(Post-hoc Dunn test with Bonferroni correction)** | | |
| --- | --- | --- |
| **Median DDS of children (24-hour recall data)** | | |
| Visit | Comparison arms | p-value |
| Week 6 | Arm A vs SOC | 0.002 |
|  | Arm B vs SOC | 0.002 |
|  | Arm B vs Arm A | 1.00 |
| Week 12 | Arm A vs SOC | 0.001 |
|  | Arm B vs SOC | 0.001 |
|  | Arm B vs Arm A | 1.00 |
| **Median DDS of children (7-day food frequency data)** | | |
| Week 1 | Arm A vs SOC | <0.001 |
|  | Arm B vs SOC | <0.001 |
|  | Arm B vs Arm A | 0.469 |
| Week 6 | Arm A vs SOC | <0.001 |
|  | Arm B vs SOC | <0.001 |
|  | Arm B vs Arm A | 1.00 |
| Week 12 | Arm A vs SOC | <0.001 |
|  | Arm B vs SOC | <0.001 |
|  | Arm B vs Arm A | 1.00 |
| **Visits with significant results on the ANOVA test**  **(Post-hoc Turkey HSD test)** | | |
| **Caregiver knowledge scores** | | |
| Week 6 | Arm A vs SOC | 0.004 |
|  | Arm B vs SOC | 0.009 |
|  | Arm B vs Arm A | 0.973 |
| Week 12 | Arm A vs SOC | <0.001 |
|  | Arm B vs SOC | 0.036 |
|  | Arm B vs Arm A | 0.413 |
